# The interplay between probiotics and mast cells in gut inflammation: a mini-review

**DOI:** 10.3389/fcimb.2026.1772010

**Published:** 2026-04-01

**Authors:** Maria Fernanda Herrera-Saldivar, Norma Carolina Hernandez-Bautista, Juan Manuel Quiroga-Garza, Jose Manuel Vazquez-Guillen, Rommel Chacon-Salinas, Maurilia Rojas-Contreras, Ricardo Vazquez-Juarez, Diana Resendez-Perez, Reyes S. Tamez-Guerra, Cristina Rodriguez-Padilla

**Affiliations:** 1 Universidad Autónoma de Nuevo León, Facultad de Ciencias Biológicas, Laboratorio de Inmunología y Virología, San Nicolás de los Garza, Nuevo León, Mexico; 2 Universidad Autónoma de Nuevo León, Facultad de Ciencias Biológicas, Departamento de Biología Celular y Genética, San Nicolás de los Garza, Nuevo León, Mexico; 3 Instituto Politécnico Nacional, Escuela Nacional de Ciencias Biológicas, Departamento de Inmunología, Ciudad de México, Mexico; 4 Universidad Autónoma de Baja California Sur, Laboratorio de Ciencia y Tecnología de los Alimentos, La Paz, Baja California Sur, Mexico; 5 Centro de Investigaciones Biológicas del Noroeste, S.C., Laboratorio de Genómica y Bioinformática, La Paz, Baja California Sur, Mexico

**Keywords:** gastrointestinal diseases, inflammation, intestinal barrier function, mast cells, probiotics

## Abstract

Mast cells (MCs) are myeloid-derived immune cells that differentiate in peripheral tissues, where they are strategically located near epithelial surfaces. In the gastrointestinal tract, MCs regulate immune response through the release of mediators such as histamine, cytokines, and proteases; however, their excessive activation promotes inflammation, increases intestinal permeability, and contributes to disorders such as food allergies, inflammatory bowel disease, and chronic intestinal inflammation. Probiotics are live microorganisms that confer health benefits to the host when administered in adequate amounts and play a key role in gut immune regulation by modulating the microbiota, reinforcing epithelial barrier integrity, and promoting anti-inflammatory responses. Although MCs and probiotics have been extensively studied independently, their functional interplay in gut health and disease remains poorly defined. Here, we propose that probiotic-mast cell interactions represent an underexplored immunoregulatory axis in intestinal homeostasis. This mini-review synthesizes current evidence on the crosstalk between probiotics and MCs, highlighting the limited mechanistic understanding of these interactions and its potential therapeutic implications.

## 1 Introduction

Mast cells (MCs) originate in the bone marrow from the myeloid lineage. They circulate in the blood as immature precursors and complete their differentiation in peripheral tissues, where they acquire specific phenotypic characteristics shaped by local extracellular matrix proteins, adhesion molecules, chemokines, and cytokines (Lichterman and Reddy, 2021). As a result, mature MCs are heterogeneous and display tissue-specific profiles in mediator content and functional properties (Traina, 2021).

MCs are widely distributed in mucosal and connective tissues, including the skin and the respiratory, gastrointestinal, and urogenital tracts, positioning them at key interfaces with the external environment. Under physiological conditions, they act as first-line defenders against physical, chemical, or microbial threats (Sobiepanek et al., 2022; Dileepan et al., 2023). Upon activation, MCs orchestrate allergic and inflammatory responses through the release of a broad panel of mediators and cytokines, thereby contributing to both host protection and the regulation of local immune homeostasis (Krystel-Whittemore et al., 2016). In the gastrointestinal tract, MCs play a crucial role in maintaining epithelial integrity, regulating tissue perfusion, and coordinating motility to facilitate the clearance of harmful agents (Papa et al., 2025). They also participate in coagulation, modulation of vascular permeability, peristalsis, nociception, and immune responses to pathogens (Van Diest et al., 2012; Mai et al., 2021). These functions contribute to the fine balance between innate and adaptive immunity (Katsoulis-Dimitriou et al., 2020).

Activation of intestinal MCs leads to the release of pro-inflammatory mediators, including histamine, cytokines, and proteases such as tryptase and chymase, which amplify inflammation, increase intestinal permeability, and promote conditions such as food allergies, inflammatory bowel disease (IBD), irritable bowel syndrome (IBS), and chronic intestinal inflammation (Atiakshin et al., 2019; Theoharides et al., 2023). These mediators act at multiple levels of the inflammatory cascade by modulating vascular flow and permeability, recruiting immune cells, and shaping downstream cytokine networks (Czarnetzki et al., 1995). Given the central role of mast cell-derived pro-inflammatory mediators in gastrointestinal disorders described above, interest has grown in therapeutic strategies aimed at modulating these pathways. Among these, probiotics have emerged as promising candidates, particularly in IBD, where they have been shown to alleviate symptoms and improve quality of life. Their beneficial effects are attributed to their ability to modulate gut microbiota composition, regulate immune responses, reinforce epithelial barrier function, and reduce intestinal inflammation (Vallejos et al., 2025; Zhang et al., 2025).

**Figure 1:**
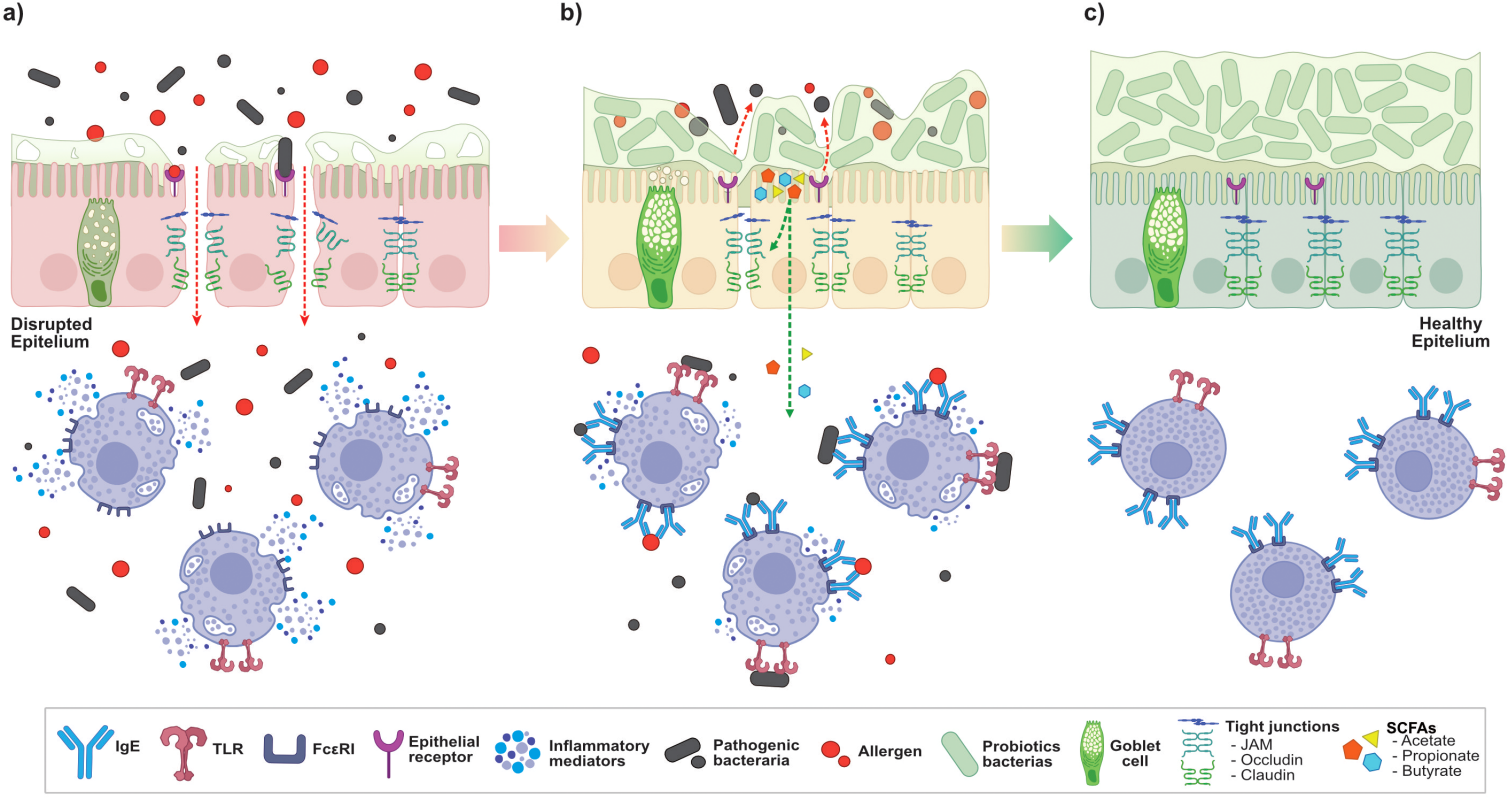
Probiotic-mediated restoration of intestinal barrier function and modulation of mast cells (MCs) activation. **(A)** *Disrupted epithelium*. Under pathological conditions, intestinal barrier dysfunction is characterized by thinning and discontinuity of the mucus layer, disorganization of tight junction proteins, and increased epithelial permeability. Pathogenic bacteria can penetrate the weakened mucus and interact with epithelial receptors, facilitating allergen translocation into the lamina propria. This promotes local inflammation through the release of pro-inflammatory mediators and activation of MCs via both IgE-dependent and IgE-independent pathways. **(B)** *Probiotic intervention*. Probiotics compete with pathogenic bacteria for epithelial adhesion sites and contribute to restoration of barrier integrity. They produce short-chain fatty acids (SCFAs), which act on epithelial and immune cell receptors to enhance tight junction protein expression and reinforce epithelial cohesion. Concurrently, probiotic-derived signals attenuate mast cell activation and bias intracellular signaling away from excessive pro-inflammatory mediator release. **(C)** *Restored homeostasis*. Following probiotic-mediated modulation, the mucus layer regains structural continuity, tight junction organization is restored, and spatial segregation between microbiota and the epithelial surface is maintained. Controlled MCs activation limits inflammatory mediator release, thereby promoting intestinal immune homeostasis.

Probiotics are live microorganisms that confer health benefits to the host when administered in adequate amounts (Suez et al., 2019). To exert their beneficial effects in the gut, orally administered probiotic strains must survive harsh gastric conditions and bile exposure to reach the small and large intestines in a viable state (Bengoa et al., 2018). This requires tolerance to acid pH and bile salts, as well as the capacity to adhere to the intestinal epithelium (Sarita et al., 2025). Patients with IBD commonly exhibit dysbiosis and impaired epithelial barrier function, both of which contribute to persistent inflammation and disease progression (Yu, 2018). In these inflammatory conditions, probiotics can restore microbial balance, reinforce the epithelial integrity, and promote the production of anti-inflammatory cytokines, thereby limiting the release of pro-inflammatory mediators (**Figure 1**). Although MCs and probiotics have been studied separately in relation to gut health and disease, their combined roles remain poorly understood. This mini-review summarizes current evidence on probiotic-mast cell interaction, emphasizing the limited mechanistic understanding of their interplay and highlighting this gap as a critical area for future research.

## 2 Mast cells in gut health and disease

MCs exhibit remarkable heterogeneity, with their phenotypes and functional responses shaped by the surrounding tissue microenvironment and by the nature of the mediators they release (West and Bulfone-Paus, 2022). Upon activation, MCs initiate a biphasic secretory process. Preformed mediators stored in cytoplasmic granules, such as histamine, heparin, serotonin, and the proteases chymase and tryptase, are rapidly released to trigger immediate responses. In contrast, newly synthesized mediators, including lipid-derived molecules, cytokines, and chemokines, are produced after stimulation and sustain the inflammatory response for several hours following the initial activation (Elieh Ali Komi et al., 2020).

Human MCs are commonly classified according to their protease content. The MC_T_ subset is characterized by high tryptase levels with little or no chymase, whereas the MC_TC_ subtype contains tryptase, chymase, and carboxypeptidase. A third subset, MC_C_, which expresses chymase but little or no tryptase, has also been described, although it appears to be relatively rare (Wouters et al., 2016). The specific protease profile of each mast cell subset critically determines its biological functions during activation and degranulation (Fernandez-Blanco et al., 2015). In the gastrointestinal tract, MC_T_ cells predominate within the intestinal mucosa, while MC_TC_ cells are more abundant in the submucosa (Putro et al., 2024). Most intestinal MC_C_ reside in the lamina propria of the duodenal and colonic mucosa, where they are strategically positioned near nerves, epithelial cells, and microvasculature (Albert-Bayo et al., 2019; Ribatti, 2024). This anatomical localization allows MC_C_ to function as frontline sentinels, essential for maintaining homeostasis and coordinating local inflammatory processes (Molfetta and Paolini, 2023).

MCs contribute to immune surveillance by detecting microbial components through pattern recognition receptors (PRR), including C-type lectin-like receptors (CLR), retinoic acid-inducible gene-I-like receptors (RLR), nucleotide-binding oligomerization domain-like receptors (NLR), and Toll-like receptors (TLR). These receptors allow MCs to sense both pathogen-associated and damage-associated molecular patterns and initiate appropriate immune responses (Agier et al., 2018). In the healthy gut, intestinal MCs express high levels of TLR, particularly TLR-2 and TLR-4, which recognize components from Gram-positive and Gram-negative bacteria, respectively. Activation of TLR-2 promotes MCs degranulation followed by cytokine production, while TLR-4 stimulation induces cytokine release without triggering degranulation (Molfetta et al., 2025). These signaling pathways ultimately lead to the recruitment of immune cells and amplification of local defense mechanisms (Lakatos and Rosta, 2025).

Intestinal MCs also contribute to adaptive immunity through immunoglobulin E (IgE)-mediated activation. They express the high-affinity IgE receptor FcϵRI, and when allergen exposure leads to cross-linking of FcϵRI-bound IgE, MCs become activated and initiate degranulation (Fukatsu et al., 2021). This mechanism is central in food allergies, where IgE-mediated mast cell activation can result in localized gastrointestinal symptoms or systemic reactions such as anaphylaxis (Oettgen, 2023).

MCs are essential for maintaining gut homeostasis. By releasing mediators in a controlled manner, they regulate mucosal permeability and epithelial integrity (Bilgic et al., 2026). Gastrointestinal MCs modulate intercellular junctions and transiently increase tight junction permeability through activation of protease-activated receptors, promoting fluid secretion and peristaltic activity to facilitate the removal of potentially harmful agents (Jacob et al., 2005). Under physiological conditions, this controlled increase in permeability allows minimal amounts of antigens and bacteria to traverse the epithelium, enabling regulated interactions with innate and adaptive immune cells without compromising barrier function (Keita and Söderholm, 2018).

However, dysregulated MCs activity can significantly disturb gut function. Increase in MCs number or excessive degranulation leads to enhanced pain perception, intestinal hyperpermeability, altered motility, and abnormal secretory responses (McClain et al., 2020). Abnormal mast cell activation has associated with multiple gastrointestinal disorders, including celiac disease, IBS, IBD (Crohn’s disease and ulcerative colitis), and food allergies (Bischoff, 2016; Dvornikova et al., 2023).

## 3 Probiotics as modulators of gut immunity

Given the central role of dysregulated mast cell activity in gastrointestinal disorders, strategies to modulate their function are of therapeutic interest. Probiotics, with their established immunomodulatory properties, represent a potential avenue for such modulation, prompting an examination of their direct and indirect effects on MCs. Early definitions of probiotics described them as substances produced by bacteria that stimulate the growth of other microorganisms (Wieërs et al., 2020). In 2002, the Food and Agriculture Organization (FAO) and the World Health Organization (WHO) refined this concept, defining probiotics as live microorganisms that, when administered in adequate amounts, confer a health benefit on the host (Suez et al., 2019). Most probiotic strains belong to bacterial species that naturally inhabit the human gastrointestinal tract (Wilkins and Sequoia, 2017). Among these, *Lactobacillus* and *Bifidobacterium* are the genera most commonly used in probiotic products (Huys et al., 2013). Probiotic bacteria exert their beneficial effects through multiple mechanisms, including competitive exclusion of pathogens by preventing their adhesion to the intestinal epithelium, inhibition of pathogenic growth through competition for essential nutrients, and the production of bioactive compounds such as short-chain fatty acids (SCFAs) and bacteriocins (Latif et al., 2023). SCFAs are metabolic products derived from the fermentation of non-digestible dietary fiber, whereas bacteriocins are a heterogeneous group of antimicrobial peptides with activity against pathogenic microorganisms (Thoda and Touraki, 2023).

Probiotics are key modulators of the intestinal immune environment. They adhere to epithelial cells and activate them through PRR-mediated signaling. This interaction induces the release of cytokines that promote the differentiation and expansion of regulatory T (Treg) cells, which play a central role in maintaining immune tolerance and mucosal homeostasis (Mazziotta et al., 2023). Treg cells efficiently suppress excessive immune activation and prevent uncontrolled inflammatory responses in the colon, largely through the production of anti-inflammatory cytokines such as interleukin-10 (IL-10) (Aghamohammad et al., 2022). Thus, the immunomodulatory effects of probiotics appear to be largely mediated by their capacity to promote anti-inflammatory cytokine responses within the gut (Cristofori et al., 2021).

In addition to their effects on T-cell-mediated immunity, probiotics influence humoral immune responses at the mucosal level. They promote immunoglobulin class switching in mature B cells within Peyer’s patches toward secretory immunoglobulin A (IgA)-producing plasma cells (Hardy et al., 2013). Secretory IgA contributes to intestinal homeostasis by blocking pathogen adherence to epithelial receptors, neutralizing bacterial toxins, and preserving mucosal barrier integrity (Rousseaux et al., 2023). Furthermore, specific probiotic strains can enhance intestinal epithelial function by modulating mucin production and tight junction protein expression, thereby strengthening barrier integrity, and indirectly shaping gut immune responses (La Fata et al., 2018). *In vitro* and *in vivo* studies have shown that *Lactobacillus plantarum* 299v, *Escherichia coli* Nissle 1917, *Lactobacillus casei* GG, and the probiotic mixture VSL#3 increase the expression of genes involved in mucin production (Mack et al., 1999; A. et al., 2002; Caballero-Franco et al., 2007; Hafez, 2012). Additionally, *Escherichia coli* Nissle 1917, *Lactobacillus rhamnosus* GG, *Lactobacillus casei* DN-114001, *Bifidobacterium infantis* (from VSL#3), and *Lactobacillus plantarum* strains MB452, WCFS1, and CGMCC No.1258 have been shown to modulate tight junction protein expression and distribution in a strain-dependent manner, ultimately reducing epithelial permeability (**Table 1**) (Ukena et al., 2007; Zyrek et al., 2007; Ewaschuk et al., 2008; Johnson-Henry et al., 2008; Anderson et al., 2010; Karczewski et al., 2010; Qin et al., 2009; Parassol et al., 2005).

**Table 1:** Evidence of interactions between mast cells (MCs) and probiotics: probiotic strains, experimental models, and reported effector mechanisms.

Mechanism	Probiotic strain	Model of study	Reference
Enhance the expression of mucin-related genes			
	*Lactobacillus plantarum* 299v	Human colorectal adenocarcinoma (HT-29)	Mack et al., 1999
	*Lactobacillus casei* GG	Human colorectal adenocarcinoma (Caco-2)	Mattar et al., 2002
	VSL#3 multi-strain formulation	Wistar rats	Caballero-Franco et al., 2007
	*Escherichia coli* Nissle 1917	Human colorectal adenocarcinoma (HT-29)	Hafez, 2012
Regulate tight junction proteins			
	*Lactobacillus casei* DN-114001	Human colon carcinoma (T84)	Parassol et al., 2005
	*Escherichia coli* Nissle 1917	BALB/c mice	Ukena et al., 2007
	*Escherichia coli* Nissle 1917	Human colon carcinoma (T84)	Zyrek et al., 2007
	*Lactobacillus rhamnosus* GG	Canine kidney (MDCK.I) Human colon carcinoma (T84)	Johnson-Henry et al., 2008
	*Bifidobacterium infantis* (from VSL#3 multi-strain formulation)	Human colon carcinoma (T84)	Ewaschuk et al., 2008
	*Lactobacillus plantarum* CGMCC No.1258	Human colorectal adenocarcinoma (Caco-2)	Qin et al., 2009
	*Lactobacillus plantarum* MB452	Human colorectal adenocarcinoma (Caco-2)	Anderson et al., 2010
	*Lactobacillus plantarum* WCFS1	Randomized human crossover study and Human colorectal adenocarcinoma (Caco-2)	Karczewski et al., 2010
Attenuation of IgE-dependent mast cell activation			
	*Lactobacillus rhamnosus* Lc705	Human peripheral blood mononuclear-derived mast cells	Oksaharju, 2011
	*Lactobacillus casei* Shirota *Lactobacillus salivarius* HMI001	C3H/HeOuJ mice	Meijerink et al., 2012
	*Lactobacillus paracasei* S1, S8, S21, S25, S31, and S40	Mouse bone marrow-derived mast cells	Cassard et al., 2016
	*Lactobacillus rhamnosus* GG *Lactobacillus plantarum* CJLP133 and CJLP243	BALB/c mice	Hyung et al., 2017
	*Bifidobacterium longum*	BALB/c mice	An et al., 2022
Inhibition of mast cell degranulation by IgE-independent-mechanism			
	VSL#3 multi-strain formulation	Sprague-Dawley rats	Li et al., 2020
	*Lactobacillus casei* ATCC 393	C57BL/6 mice	Xu et al., 2020
	*Lactiplantibacillus plantarum* HD02, MD159, and MD161 *Limosilactobacillus fermentum* MD58 *Lactobacillus gasseri* MK03	Rat basophils-derived mast cells (RBL-2H3)	Kim et al., 2024
	*Lactobacillus plantarum* ZYC501	C57BL/6 mice	Yao et al., 2025
Alteration of mediator release profiles			
	*Lactobacillus casei* ATCC 393	C57BL/6 mice	Song et al., 2021
	*Lactobacillus rhamnosus* LR32 *Bifidobacterium lactis* BL04 *Bifidobacterium longum* BB536	Human mast cell line (HMC-1.2) cultivated with human colorectal adenocarcinoma (Caco-2 and HT-29)	di Vito et al., 2023

## 4 Interplay between probiotics and mast cells

### 4.1 Modulation of IgE-dependent activation

Accumulating evidence indicates that probiotics can modulate mast cell activation by interfering with immunoglobulin E (IgE)-dependent signaling pathways. In allergic individuals, allergen-specific T-helper 2 responses promote IgE production, which binds to FcϵRI on MCs. Crosslinking of FcϵRI-bound IgE triggers degranulation and the release of mediators responsible for allergic symptoms (Van Esch et al., 2016). Targeting this pathway therefore represent a critical mechanism for controlling MCs-driven allergic activation. Several *in vitro* studies demonstrate that probiotic strains directly influence FcϵRI-mediated responses. For instance, individual *Lactobacillus paracasei* strains differentially inhibit IgE-induced activation of murine MCs, underscoring the strain-dependent nature of probiotic effects (Cassard et al., 2016). Similarly, *Lactobacillus rhamnosus* GG (ATCC 53103) and Lc705 (DSM 7061) suppress the expression of FCER1A and HRH4 in human MCs, genes associated with receptor signaling and histamine responsiveness (Oksaharju, 2011). Together, these findings suggest that certain probiotics may act upstream of degranulation by modulating FcϵRI signaling components rather than merely limiting mediator release.

*In vivo* evidence further supports these findings. In murine models of food allergy, *Lactobacillus casei* Shirota and *Lactobacillus salivarius* HMI001 reduced IgE levels and decreased markers of mast cell degranulation (Meijerink et al., 2012). Likewise, *Lactobacillus rhamnosus* GG and *Lactobacillus plantarum* strains CJLP133 and CJLP243, isolated from kimchi, attenuated food allergy symptoms in BALB/c mouse model by suppressing mast cell activation (Hyung et al., 2017).

In experimental allergy models, probiotic supplementation may also enhance immunotherapeutic approaches. In a BALB/C mouse model of food allergy, *Bifidobacterium longum* acted as an effective co-adjuvant when combined with anti-IgE antibody therapy, leading to reduced effector cell numbers, decreased circulating IgE levels, and attenuated mast cell degranulation (An et al., 2022). These findings suggest that probiotics may not only modulate IgE-dependent activation directly but also synergize with IgE-targeted therapies, further supporting their potential role in regulating FcϵRI-driven mast cell responses.

### 4.2 Regulation of MCs degranulation via non-IgE pathways

Beyond FcϵRI signaling, MCs can degranulate through IgE-independent mechanisms involving complement components, TLR ligands, neuropeptides, cytokines, and microbial products (Yu et al., 2016). Modulation of these alternative pathways may be particularly relevant in inflammatory and infectious contexts.

Emerging *in vivo* evidence suggests that probiotics can attenuate mast cell hyperactivation independently of allergen-specific IgE signaling. In a murine model of IBS, administration of *Lactobacillus plantarum* ZYC501 combined with galacto-oligosaccharides reduced histamine, tryptase, and prostaglandin E_2_ levels in the gut mucosa, indicating decreased MCs activation (Yao et al., 2025). Importantly, restoration of cyclooxygenase-2 levels toward baseline further supports normalization of inflammatory signaling rather than simple suppression.

Similarly, in mice with barrier dysfunction induced by enterotoxigenic *Escherichia coli* K88, *Lactobacillus casei* ATCC 393 reduced mast cell activation, potentially through modulation of TLR-2 and TLR-4 pathways (Xu et al., 2020). These findings suggest that certain probiotic strains may influence innate receptor-mediated mast cell activation, linking microbial sensing pathways with regulation of degranulation. Compared to IgE-dependent mechanisms, evidence for non-IgE pathways remains more heterogeneous and largely derived from animal models. Nonetheless, these observations broaden the conceptual framework of probiotic-MCs interaction beyond classical allergic signaling.

### 4.3 Alteration of mediator release profiles

In addition to modulating activation thresholds, probiotics appear to influence the qualitative profile of MCs mediator release. Rather than acting solely as inhibitors of degranulation, some strains reshape the inflammatory output of activated MCs.

For instance, a probiotic formulation containing *Lactobacillus rhamnosus* LR32, *Bifidobacterium lactis* BL04, and *Bifidobacterium longum* BB536 reduced interleukin-6 (IL-6) secretion in a human mast cell line co-cultured with intestinal epithelial cells, suggesting that probiotic effects may involve modulation of MCs-epithelial crosstalk (di Vito et al., 2023). Similarly, *L. casei* ATCC 393 decreased both preformed mediators (histamine, tryptase, β-hexosaminidase) and newly synthesized cytokines (TNF-α, IL-6), indicating a broad regulation of MCs secretory processes (Song et al., 2021). These findings imply that probiotics may not only suppress MCs activation but also recalibrate the inflammatory phenotype of these cells. However, mechanistic insight into how bacterial-derived metabolites or cell surfaces components influence intracellular signaling cascades within MCs remains limited.

Evidence from extra intestinal models further supports the systemic relevance of these interactions. Specific *Lactiplantibacillus plantarum* strains attenuate mast cell degranulation in an atopic dermatitis model (Kim et al., 2024). Also, the multi-strain formulation VSL#3 reduces mast cell degranulation in visceral hypersensitivity mouse models (Li et al., 2020). These observations suggest that probiotic-mast cell interactions may extend beyond the gut, although translation to human disease requires further validation.

## 5 Conclusion

Although MCs and probiotics have been extensively studied independently in the context of gut health, the biological significance of their interaction remains incompletely understood. This mini-review highlights emerging evidence that specific probiotic strains modulate MCs activation and effector functions in a strain-dependent manner, promoting immune tolerance and limiting excessive inflammation. However, most available data derive from indirect or experimental observations, leaving the direct mechanistic crosstalk between probiotics and MCs insufficiently characterized. We propose that probiotic-mast cell interactions constitute a previously underappreciated immunoregulatory axis within the intestinal microenvironment. Defining this axis at the molecular and cellular levels may reshape current understanding of microbiota-driven immune regulation and foster the development of targeted probiotic strategies for MCs-mediated gastrointestinal disorders. Future studies should prioritize mechanistic approaches capable of directly interrogating these interactions. Co-culture systems integrating human MCs and intestinal epithelial cells would more accurately model the gastrointestinal niche. *In vivo* models, such as LPS-induced systemic inflammation with probiotic pretreatment, may allow direct assessment of probiotic-mediated modulation of MCs degranulation and cytokine release. Clinical correlative studies linking patient mast cell transcriptional profiles to probiotic responsiveness could identify predictive biomarkers, while metabolomic analyses aimed to identifying key bacterial metabolites involved in MCs modulation will be essential for defining the underlying regulatory pathways.
